# A systematic review and meta-analysis of outpatient treatment for acute diverticulitis

**DOI:** 10.1007/s00384-018-3015-9

**Published:** 2018-03-12

**Authors:** S. T. van Dijk, K. Bos, M. G. J. de Boer, W. A. Draaisma, W. A. van Enst, R. J. F. Felt, B. R. Klarenbeek, J. A. Otte, J. B. C. M. Puylaert, A. A. W. van Geloven, M. A. Boermeester

**Affiliations:** 1 Department of Surgery, Academic Medical Center, Amsterdam, the Netherlands; 2 Department of Infectious Diseases, Leiden University Medical Center, Leiden, the Netherlands; 3 Knowledge Institute of Medical Specialists, Utrecht, the Netherlands; 4 Department of Gastroenterology, VU University Medical Center, Amsterdam, the Netherlands; 5 Department of Surgery, Radboudumc, Nijmegen, the Netherlands; 6 Department of Internal Medicine, ZorgSaam Hospital, Terneuzen, the Netherlands; 7 Department of Radiology, Haaglanden Medical Center, The Hague, the Netherlands; 8 Department of Surgery, Tergooi Hospital, Hilversum, the Netherlands

**Keywords:** Acute diverticulitis, Uncomplicated diverticulitis, Outpatient treatment, Home treatment, Costs

## Abstract

**Background:**

The shift from routine antibiotics towards omitting antibiotics for uncomplicated acute diverticulitis opens up the possibility for outpatient instead of inpatient treatment, potentially reducing the burden of one of the most common gastrointestinal diseases in the Western world.

**Purpose:**

Assessing the safety and cost savings of outpatient treatment in acute colonic diverticulitis.

**Methods:**

PubMed and EMBASE were searched for studies on outpatient treatment of colonic diverticulitis, confirmed with computed tomography or ultrasound. Outcomes were readmission rate, need for emergency surgery or percutaneous abscess drainage, and healthcare costs.

**Results:**

A total of 19 studies with 2303 outpatient treated patients were included. These studies predominantly excluded patients with comorbidity or immunosuppression, inability to tolerate oral intake, or lack of an adequate social network. The pooled incidence rate of readmission for outpatient treatment was 7% (95%CI 6–9%, *I*^2^ 48%). Only 0.2% (2/1288) of patients underwent emergency surgery, and 0.2% (2/1082) of patients underwent percutaneous abscess drainage. Only two studies compared readmission rates outpatients that had similar characteristics as a control group of inpatients; 4.5% (3/66) and 6.3% (2/32) readmissions in outpatient groups versus 6.1% (4/66) and 0.0% (0/44) readmissions in inpatient groups (*p* = 0.619 and *p* = 0.174, respectively). Average healthcare cost savings for outpatient compared with inpatient treatment ranged between 42 and 82%.

**Conclusion:**

Outpatient treatment of uncomplicated diverticulitis resulted in low readmission rates and very low rates of complications. Furthermore, healthcare cost savings were substantial. Therefore, outpatient treatment of uncomplicated diverticulitis seems to be a safe option for most patients.

**Electronic supplementary material:**

The online version of this article (10.1007/s00384-018-3015-9) contains supplementary material, which is available to authorized users.

## Introduction

Diverticular disease is listed in the top five of most burdensome gastrointestinal diseases in the Western world [1]. Acute diverticulitis, the inflammatory complication of diverticular disease, accounts for approximately 150.000 emergent admissions annually [2]. Approximately one third of admitted patients with diverticulitis present with complicated disease (abscess, perforation, obstruction, fistula), two-third presents with uncomplicated disease [3, 4]. Traditionally, patients were admitted routinely for intravenous antibiotic treatment. Following several studies that reported the safety of oral antibiotic treatment, two randomized clinical trials showed that treating uncomplicated acute diverticulitis without antibiotics is safe [5, 6]. These developments opened the way for outpatient instead of inpatient treatment. Treatment of acute diverticulitis without an expensive admission may reduce the burden to the healthcare system considerably, besides potential reduction of hospital admission-related adverse effects such as delirium and hospital related infections. Previous systematic reviews on outpatient treatment of uncomplicated diverticulitis had some methodological limitations: inclusion of studies that did not study outpatient treatment specifically but based conclusions on inpatients, inclusion of studies that did not use computed tomography or ultrasound to confirm the diverticulitis diagnosis, inclusion of studies mainly reporting right-sided diverticulitis and missing several studies presumably due to narrow search strategies [7–9]. Also, several new studies have been published meanwhile (Online Resource 1). The present systematic review evaluates the safety of outpatient treatment of acute colonic diverticulitis in randomized clinical trials and observational cohort studies. Important study characteristics and their consequences will be discussed, such as generalisability of outpatient treatment protocols, potential selection bias in treatment allocation, and distinction between left- and right-sided diverticulitis.

## Methods

### Study identification

Two investigators, SD and KB, independently searched PubMed and EMBASE databases with the following search terms: diverticulitis, diverticular, ambulatory care, outpatients, ambulatory, outpatient and home (Online Resource 2). No language or date limits were applied. The last search was performed in November 2017. Reference lists of obtained articles were reviewed for omitted studies. Where there was overlap in patient cohorts of two studies, the most recent and largest study was included in this systematic review. MOOSE and PRISMA guidelines for reporting were followed [10, 11]. A review protocol for this systematic review was not published or registered before this study was undertaken.

### Study selection

Studies considered for eligibility were randomized clinical trials, prospective and retrospective cohort studies that reported outcomes of outpatient treatment of acute colonic diverticulitis, confirmed with computed tomography (CT) or ultrasound (US). Studies that included more than 20% right-sided diverticulitis were excluded. Studies that did not quantify the number of right-sided diverticulitis patients but were from Western origin were not excluded under the assumption that in the Western world the vast majority of cases (usually above 90% [12–14]) concern left-sided diverticulitis. Reviews, letters, and case reports were excluded. The two reviewers independently considered all studies retrieved from the search for eligibility against these criteria. Any disagreements in any phase of the study selection, quality assessment or data extraction were resolved through discussion.

### Quality assessment

The two reviewers (SD and KB) independently appraised each study using the Cochrane risk of bias tool for randomized controlled trials and the Newcastle Ottawa Quality Assessment Scale for cohort studies [15, 16].

### Data extraction

The two reviewers (SD and KB) independently reviewed each included article. Each reviewer independently extracted the data on a predefined evidence table, after which the two tables were compared. Data collected from each paper was study design and setting; diagnostic modality (CT and/or ultrasound); in- and exclusion criteria for the study and, if different, for outpatient treatment; proportion of left- or right-sided diverticulitis; description of outpatient treatment protocol; criteria for assignment to outpatient or inpatient group; reported outcome measures and results.

### Outcome measures

Primary outcome measure was rate of readmission after start of outpatient or inpatient treatment. Secondary outcome measures were need for emergency surgery, the need for percutaneous abscess drainage, and costs.

### Statistical analysis

The incidence rates of readmission in the outpatient groups of the included studies were pooled and displayed using a forest plot and a random effects model. Statistical heterogeneity was assessed using *χ*^2^ and *I*^2^. Statistical analyses were conducted using RStudio (RStudio Inc., Boston, MA, USA).

## Results

### Systematic review

**Fig. 1:**
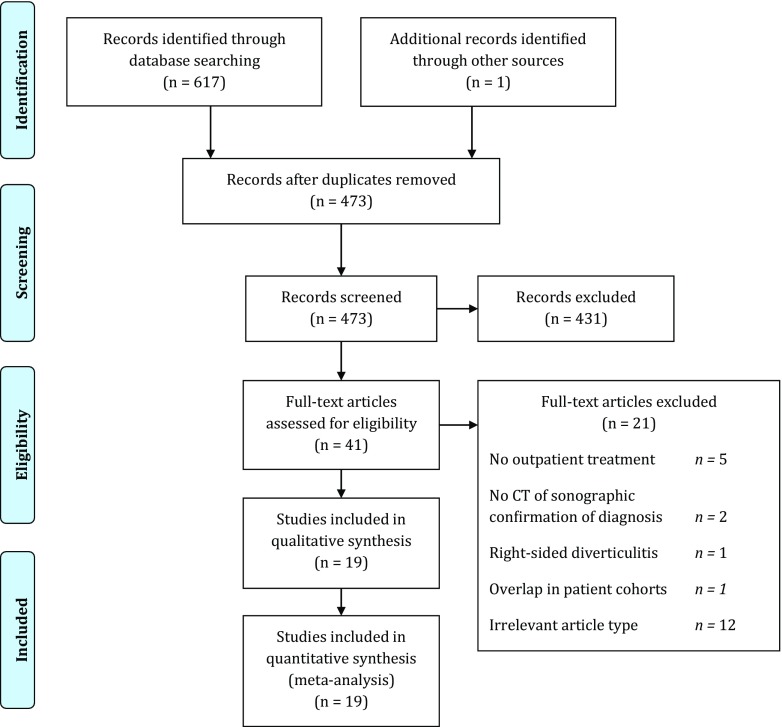
PRISMA flow diagram [11]

The search retrieved 617 studies, one additional study was identified through cross-referencing. After removal of 145 duplicates, 473 articles were screened. Based on title and abstract, 431 articles were excluded and 41 full texts were assessed for eligibility. After applying in- and exclusion criteria, 19 studies were included in this review. Figure 1 shows the results of the search strategy. Online Resource 3 shows the reasons for exclusion of 21 full text articles.

### Study characteristics

Table 1 shows the summary of included studies. One randomized clinical trial [17], 10 prospective cohort studies [18–27] and 8 retrospective cohort studies [28–35] were included. Most studies (*n* = 12) were performed in Spain, the other studies were performed in Finland, Sweden, the Netherlands, Switzerland and the USA. All but one study used CT to confirm the diverticulitis diagnosis; a Dutch study [35] used either CT or ultrasonography. Although all studies included patients that received outpatient treatment, different treatment protocols were used. In most studies, outpatient treatment consisted of ambulatory treatment at home with oral antibiotics and a liquid diet during the first couple of days followed by outpatient clinic visits after 4 to 7 days. Five studies did not define the outpatient treatment protocol. Three studies specifically stated that all patients were treated without antibiotics [19–21]. Most studies selected patients as outpatient treatment candidates based on patient characteristics (such as absence of comorbidities or immunosuppressed state), clinical condition (such as having uncomplicated diverticulitis and ability to tolerate oral intake) and patients’ social environment (adequate family and social network). Importantly, seven studies [22, 28, 29, 31, 33–35] also included patients with diverticular abscesses as candidates for outpatient treatment. Although most studies used outpatient treatment protocols that could be used in almost all hospitals (ambulatory treatment at home with an outpatient clinic visit after 4 to 7 days), 3 studies treated their patients in a ‘hospital at home unit’ or ‘home care unit’ [26, 27, 33]. In case of the ‘hospital at home unit’ patients were treated at home with a nurse visiting all patients daily and a physician visiting all patients 2 to 3 times a week, while all patients were treated with intravenous antibiotics [26, 27]. The study that treated their patients in a ‘home care unit’ did not provide a detailed description of this treatment strategy [33]. However, the routine intravenous antibiotic treatment suggests a protocol similar to the ‘hospital at home unit’. The two ‘hospital at home unit’ studies also included a different type of patient, as these 2 studies included patients with present comorbidity [27] or only patients older than 70 years [26].

**Table 1:** Summary of included studies and readmission rates

		Inclusion outpatients			Treatment			
Study	Study design	Abscess	Comor-bidity	Left-sided	Antibiotics	First follow-up after	Readmission outpatient	Readmission inpatient
Alonso 2010^18^	Pros	No	No	100%	Yes	4–7 days	3% (2/70)	–
Biondo 2014^17^	RCT	No	No	100%	Yes	Daily	4.5% (3/66)	6.1% (4/66)
Estrada 2016^19^	Pros	No	No	100%	No	48 h	11.1% (4/36)	33.3% (3/9)
Etzioni 2010^28^	Retro	Yes	NR	NR	NR	NR	5.6% (39/693)	–
Isacson 2015^20^	Pros	No	No	100%	No	1 week	2.3% (4/155)	–
Joliat 2017^29^	Retro	Yes	Yes	96%	Yes	NR	10.2% (10/98)	32.0% (54/169)
Lorente 2013^30^	Retro	No	No	NR	Yes	4–7 days	5.6% (5/90)	4.3% (2/46)
Lutwak 2012^32^	Retro	No	No	NR	Yes	NR	14.3% (3/21)	0.0% (0/21)
Mali 2016^21^	Pros	No	No	94%	No	24-48 h	2.9% (4/140)	–
Martin Gil 2009^22^	Pros	Yes	No	NR	Yes	10 days	5.4% (4/74)	–
Mora 2017^23^	Pros	No	No	NR	Yes	2 weeks	8.7% (22/254)	–
Moya 2012^24^	Pros	No	No	84%	Yes	4 days	6.3% (2/32)	0.0% (0/44)
Moya 2016^31^	Retro	Yes	No	95%	Yes	4 days	8.0% (18/224)	–
Pelaez 2006^25^	Pros	No	No	100%	Yes	4 days	5.0% (2/40)	–
Rodriguez 2010^27^	Pros	No	Yes	NR	Yes	Daily	0.0% (0/24)	–
Rodriguez 2013^26^	Pros	No	Yes	NR	Yes	Daily	0.0% (0/34)	–
Rueda 2012^33^	Retro	Yes	No	NR	Yes	NR	21.1% (8/38)	27.8% (5/18)
Sirany 2017^34^	Retro	Yes	Yes	96%	Yes	NR	12.5% (12/96)	15.3% (22/144)
Ünlü 2013^35^	Retro	Yes	Yes	100%	Some^a^	1 week	8.5% (10/118)	–

*Pros*, prospective cohort study; *Retro*, retrospective cohort study; *RCT*, randomized controlled trial; NR, not reported

^a^7 (6%) of 118 patients were treated with antibiotics

Thirteen studies [17, 19, 21–24, 26, 29, 30, 32–35] compared results from the outpatient treatment group with a reference group consisting of admitted patients. However, in 11 out of these 13 studies these reference patients were admitted because of the presence of one or more exclusion criteria for outpatient treatment or because of a decision by the treating physician based on the clinical condition of the patient, and thereby not strictly comparable to those treated as outpatients. Only in a randomized clinical trial [17] (randomizing between in- or outpatient treatment of uncomplicated diverticulitis patients) and a prospective cohort study [24] (selecting patients based on the time period they were treated in; before or after a change in hospital guidelines), a reliable comparison of outcomes could be made. All 19 studies reported rates of readmission, 16 studies [17–22, 24–27, 30–32, 34–36] reported rates of need for emergency surgery, 15 studies [17–20, 22, 24–27, 30–32, 34–36] reported need for percutaneous abscess drainage, and 5 studies [17, 22, 24, 26, 30] reported healthcare costs. All study characteristics are shown in Online Resource 4.

### Population characteristics

A total of 2303 patients that received outpatient treatment were included. Rates of need for emergency surgery were reported in 16 studies including a total of 1288 patients and need for percutaneous abscess drainage in 15 studies including a total of 1082 patients.

### Critical appraisal

The only randomized controlled trial [17] suffered possible selection bias and performance bias due to presumably not using opaque and sequentially numbered envelopes and the lack of blinding of participants and personnel for treatment allocation (Online Resource 5). The 18 observational studies mainly suffered possible bias due to the lack of representative control groups, the selection of patients for treatment allocation, no adjustment for confounders and the lack of description of the follow-up (see Online Resource 6).

### Readmission

**Fig. 2:**
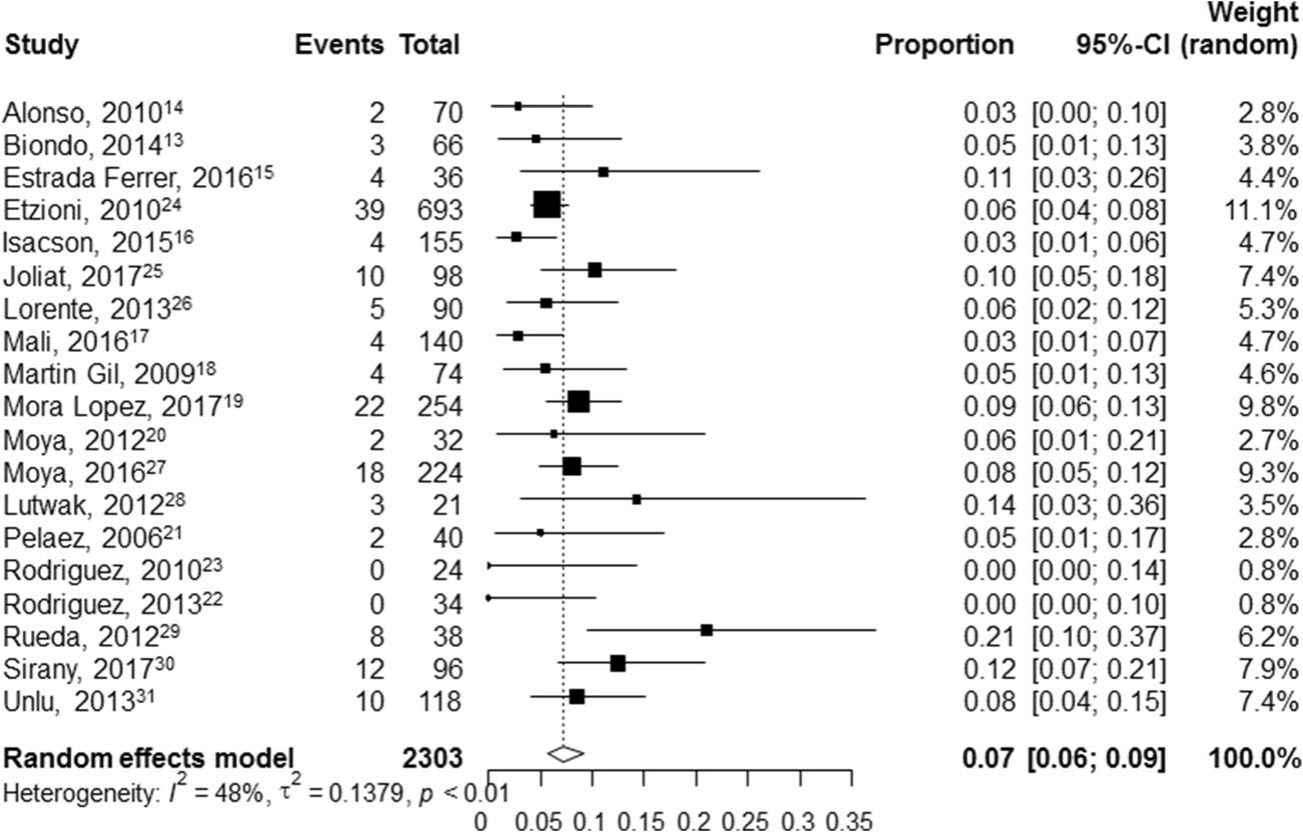
Forest plot of pooled incidence rate of readmission in patients that received outpatient treatment

All 19 studies reported rates of readmission (Table 1). Although, one retrospective cohort study [28] reported a combined endpoint of non-elective readmission or emergency department evaluation instead of solely readmission. The aforementioned two studies with representative control groups found a 4.5% (3/66) and 6.3% (2/32) readmission rate in the outpatient group versus a 6.1% (4/66) and 0.0% (0/44) readmission rate in the inpatient group (*p* = 0.619 and *p* = 0.174) respectively) [17, 24]. The pooled incidence rate of readmission in the outpatient treatment group from all 19 studies was 7% (95% CI 6–9%) (Fig. 2). When only the rates of readmission in outpatient treatment groups from studies that employed a representative ambulatory home treatment protocol (excluding 3 aforementioned studies [26, 27, 33]) were assessed, the pooled incidence rate did not change (pooled readmission rate 7%; 95% CI 6–9%, *I*^2^ 35%) (see Online Resource 7). Pooling the rates of readmission from the 6 studies that solely included left-sided diverticulitis yielded comparable results (pooled readmission rate 6%; 95% CI 3–9%, *I*^2^ 32%) (see Online Resource 8).

### Need for emergency surgery or percutaneous abscess drainage

A total of 16 studies reported rates of need for emergency surgery in the group of patients that received outpatient treatment. In all 16 studies combined, only 2 (0.2%) of 1288 patients underwent emergency surgery. The need for percutaneous abscess drainage was reported by 15 studies in which only 2 (0.2%) patients underwent percutaneous abscess drainage from a combined total of 1082 patients. No mortality occurred in all studies.

### Costs

Five studies reported a comparison of healthcare costs between outpatient and inpatient treatment. No additional cost components such as production loss were reported. Outpatient treatment resulted in average cost savings that ranged from 42 to 82% when compared to inpatient treatment in 4 studies (Table 2). One study only reported a cost saving of €1368.00 for outpatient treatment without reporting the absolute costs in each treatment group [26].

**Table 2:** Average costs (in Euros) of patients that received outpatient or inpatient treatment

	Outpatient treatment	Inpatient treatment	Savings in euros	Savings in percentages
Biondo, 2014^17^	547	1672	1125	67%
Lorente, 2013^30^	882	2376	1494	63%
Martin Gil, 2009^22^	1280	2192	912	42%
Moya, 2012^24^	347	1945	1598	82%
Rodriguez, 2013^26^	NR	NR	1368	NR

*NR*, not reported

## Discussion

The results of this systematic review show that outpatient treatment of uncomplicated left-sided colonic diverticulitis was associated with low readmission rates. The few readmissions were mostly caused by vomiting or persistent pain but diverticular complications were very rare. Furthermore, up to 82% potential healthcare cost savings were reported.

Since uncomplicated diverticulitis was treated with intravenous antibiotics routinely for a long time, outpatient treatment has been a subject of research specifically in the last 7 years. Outpatient treatment has not been implemented in clinical practice in most countries. From seven guidelines on the treatment of diverticular disease published in the last 5 years [37–43], only 3 make a recommendation regarding outpatient treatment [39, 41, 42]. All three suggest outpatient treatment in a selected group of patients. Since only one randomized controlled trial was published on this topic, conclusions and recommendations are also based on the available observational studies. Most of these studies have some drawbacks that potentially introduce bias. First, since the natural course of left- and right-sided diverticulitis may differ, diverticulitis literature should report the results for each subgroup separately. Unfortunately, many papers, in this review, 8 out of 19 studies, fail to report the number of right-sided diverticulitis patients in their studies. As the vast majority of patients in the Western world suffer from left-sided diverticulitis, the primary aim was to draw conclusion for this group of patients. Therefore, the meta-analysis of rates of readmission was repeated for studies including only left-sided diverticulitis, which yielded similar results. Secondly, most studies with inpatients as control group selected these patients based on lack of meeting certain in- or exclusion criteria for admission or based outpatient treatment on the clinical condition of the patients. This approach causes important selection bias and makes a representative comparison between these groups impossible without adjusting for confounders. This selection bias may not only affect the rate of readmission, but may also cause an overestimation of the reported cost savings of outpatient treatment. Only two studies could make a representative comparison; one based the treatment allocation on randomization and one study based the treatment allocation of the time period the patients were treated in, although the latter option does not rule out selection bias completely [17, 24]. Rates of readmission did not differ between the groups and were comparable with the pooled rate from all 19 studies, although the total number of patients in these 2 studies was low. Furthermore, it is questionable whether a comparison of readmission rates between in- and outpatients is highly relevant. Due to the distinct natures of these readmissions, the decision for outpatient treatment should be based on whether the absolute rate of readmission in outpatients is considered acceptable. Third, three studies employed an outpatient treatment protocol in such a way that it could not be applied in all general hospitals [26, 27, 33]. These studies treated all patients with intravenous antibiotics and daily visits by a nurse. Since most readmissions appeared to be caused by vomiting or persistent pain without diverticular complications, most patients actually requiring readmission could presumably be treated with intravenous fluids and medications covering up the true need for readmission. Fourth, almost all studies applied selection criteria for patients suitable for outpatient treatment, mostly lack of comorbidity or immunosuppression, ability to tolerate oral intake and adequate social network. Therefore, conclusions can only be drawn for this same selected group of patients. Since evidence on the safety of outpatient treatment in other patients is lacking, admission seems imperative for those patients.

This systematic review is limited by the lack of more than 1 randomized controlled trial. All other 18 studies were observational cohort studies and 8 of them were retrospective. This caused serious selection bias, which impaired the comparison between out- and inpatient treatment. Also, although one randomized controlled trial was included, the main conclusions are based on a much higher number of observational studies. Hence the quality of evidence is lower, but results are more robust. Moreover, heterogeneity in methodology in the studies further limited exact comparison between the studies. Although, subgroup analyses enabled conclusions to be made for the group of patients most of interest for the majority of clinicians in the Western world. Strengths of this systematic review are the large amount of data, yielding a more robust meta-analysis and the possibility for subgroup analyses, and the application of a random effects model to account for heterogeneity.

New randomized clinical trials are needed to confirm the results derived mostly from observational data. Also, selection of the patients suitable for outpatient treatment should be refined and the safety of outpatient treatment for patients with limited comorbidity should be considered. For now, a 7% readmission rate for outpatient treated acute diverticulitis patients seems to be an acceptable and low frequency disadvantage, in the context of very low complication rates. Therefore, outpatient treatment of uncomplicated diverticulitis patients without comorbidity and immunosuppression, being able to tolerate oral intake, and with an adequate social network seems to be a safe option. Only three of the included studies treated patients without antibiotics, but since two previous randomized clinical trials [5, 6] showed the safety of omitting antibiotics in uncomplicated acute diverticulitis, omitting antibiotics is likely to be equally safe in outpatient setting. Outpatient management of uncomplicated diverticulitis is generally safe and may have the potential to decrease the burden on healthcare costs substantially.

## Electronic supplementary material

Online Resource 1 Comparison of included studies in present study with previously published systematic reviews. (DOCX 56 kb)

Online Resource 2 Search strategy. (DOCX 22 kb)

Online Resource 3 Excluded studies. (DOCX 46 kb)

Online Resource 4 Evidence table. (DOCX 134 kb)

Online Resource 5 Risk of bias table of randomized clinical trial Biondo et al. [17] (DOCX 31 kb)

Online Resource 6 Newcastle Ottawa risk of bias table of observational cohort studies. (DOCX 54 kb)

Online Resource 7 Forest plot of pooled incidence rate of readmission in patients that received outpatient treatment excluding 3 studies that employed a deviated protocol [26, 27, 33] .(GIF 165 kb)

High resolution image (TIFF 53 kb)

Online Resource 8 Forest plot of pooled incidence rate of readmission in patients that received outpatient treatment only from studies with confirmed 100% left-sided diverticulitis. (GIF 88 kb)

High resolution image (TIFF 28 kb)
