# Supplementary material for: A systematic review and meta-analysis of outpatient treatment for acute diverticulitis
Source: Int J Colorectal Dis. 2018 Mar 12;33(5):505–12. doi: 10.1007/s00384-018-3015-9 (PMC5899114; doi:10.1007/s00384-018-3015-9)
Supplement: Supplementary file 1 — Comparison of included studies in present study with previously published systematic reviews. (DOCX 56 kb) [file 384_2018_3015_MOESM1_ESM.docx]

**Online Resource** 1. Comparison of included studies in present study with previously published systematic reviews.

|  | Van Dijk 2018 | Jackson 2014 | Balasubra 2016 | Sanchez 2016 | Reason for exclusion |
| --- | --- | --- | --- | --- | --- |
| Alonso 2010 | X | X | X | X |  |
| Al Sahaf 2008 |  | X |  |  | No outpatient treatment |
| Biondo 2014 | X |  | X | X |  |
| Estrada 2016 | X |  |  |  |  |
| Etzioni 2010 | X |  |  | X |  |
| Isacson 2015 | X |  |  |  |  |
| Joliat 2017 | X |  |  |  |  |
| Lombardo 1991 |  |  |  | X | Only colonoscopy diagnosis |
| Lorente 2013 | X |  | X |  |  |
| Lutwak 2012 | X |  | X |  |  |
| Mali 2016 | X |  |  |  |  |
| Martin Gil 2009 | X | X | X | X |  |
| Mizuki 2005 |  | X |  | X | 87% right-sided diverticulitis |
| Mora Lopez 2013 |  |  | X |  | Overlap in patient cohort Mora Lopez 2017 |
| Mora Lopez 2017 | X |  |  |  |  |
| Moya 2012 | X | X | X | X |  |
| Moya 2016 | X |  |  |  |  |
| Park 2011 |  | X |  |  | Only right-sided diverticulitis |
| Pelaez 2006 | X | X | X | X |  |
| Ridgway 2009 |  | X |  |  | No outpatient treatment |
| Rodriguez 2010 | X | X |  | X |  |
| Rodriguez 2013 | X |  | X | X |  |
| Rueda 2012 | X |  | X |  |  |
| Sirany 2017 | X |  |  |  |  |
| Unlu 2013 | X |  |  | X |  |
|  | 19 studies | 9 studies | 10 studies | 11 studies |  |
