# Supplementary material for: A systematic review and meta-analysis of outpatient treatment for acute diverticulitis
Source: Int J Colorectal Dis. 2018 Mar 12;33(5):505–12. doi: 10.1007/s00384-018-3015-9 (PMC5899114; doi:10.1007/s00384-018-3015-9)
Supplement: Supplementary file 2 — Search strategy. (DOCX 22 kb) [file 384_2018_3015_MOESM2_ESM.docx]

**Online Resource 2. Search strategy.**

PubMed

diverticulitis[mesh] OR "diverticulitis, colonic"[mesh] OR diverticulitis[tiab] OR diverticular[tiab]

AND

"Ambulatory Care"[Mesh] OR "Outpatients"[Mesh] OR ambulatory[tiab] OR outpatient[tiab] OR "out-patient"[tiab] OR home[tiab]

EMBASE

1. diverticulitis/
2. “diverticulitis, colonic”/
3. diverticulitis.ti,ab,kw
4. diverticular.ti,ab,kw
5. 1 or 2 or 3 or 4
6. “ambulatory care”/
7. outpatients/
8. ambulatory.ti,ab,kw
9. outpatient.ti,ab,kw
10. out-patient.ti,ab,kw
11. home.ti,ab,kw
12. 6 or 7 or 8 or 9 or 10 or 11
13. 5 and 12
