# Supplementary material for: A systematic review and meta-analysis of outpatient treatment for acute diverticulitis
Source: Int J Colorectal Dis. 2018 Mar 12;33(5):505–12. doi: 10.1007/s00384-018-3015-9 (PMC5899114; doi:10.1007/s00384-018-3015-9)
Supplement: Supplementary file 3 — Excluded studies. (DOCX 46 kb) [file 384_2018_3015_MOESM3_ESM.docx]

**Online Resource 3. Excluded studies.**

| **Reference:** | **Reason for exclusion:** |
| --- | --- |
| **Abbas 2013^1^** | No outpatient treatment |
| **Al-Khalil 2016^2^** | Irrelevant article type |
| **Al-Sahaf 2008^3^** | No outpatient treatment |
| **Aprea 2012^4^** | No outpatient treatment |
| **Beckham 2009^5^** | Irrelevant article type |
| **Friend 2011^6^** | Irrelevant article type |
| **Gargallo 2015^7^** | Irrelevant article type |
| **Genser 2013^8^** | Irrelevant article type |
| **Isacson 2016^9^** | Irrelevant article type |
| **Kohn 2015^10^** | Irrelevant article type |
| **Lombardo 1991^11^** | No computed tomographic of sonographic confirmation of diagnosis |
| **Mizuki 2005^12^** | 87% right-sided diverticulitis |
| **Mora Lopez 2013^13^** | Overlap in patients cohorts |
| **Paolillo 2015^14^** | Irrelevant article type |
| **Papagrigoriadis 2004^15^** | No outpatient treatment |
| **Ribas 2013^16^** | Irrelevant article type |
| **Ridgway 2009^17^** | No outpatient treatment |
| **Salem 2007^18^** | No computed tomographic of sonographic confirmation of diagnosis |
| **Schmidt 2015^19^** | Irrelevant article type |
| **Steurer 2013^20^** | Irrelevant article type |
| **Tursi 2014^21^** | Irrelevant article type |

**References**

1. Abbas MA, Cannom RR, Chiu VY, et al. Triage of patients with acute diverticulitis: Are some inpatients candidates for outpatient treatment? Colorectal Disease 2013;15:451-7.

2. Al-Khalil O. [Correct and safe treatment of acute, uncomplicated diverticulitis]. Praxis 2016;105:347-8.

3. Al-Sahaf O, Al-Azawi D, Fauzi MZ, El-Masry S, Gillen P. Early discharge policy of patients with acute colonic diverticulitis following initial CT scan. International Journal of Colorectal Disease 2008;23:817-20.

4. Aprea G, Giugliano A, Canfora A, et al. Diverticular disease hospital cost impact analysis: evaluation of testings and surgical procedures in inpatient and outpatient admissions. BMC surgery 2012;12 Suppl 1:S3.

5. Beckham H, Whitlow CB. The medical and nonoperative treatment of diverticulitis. Clinics in colon and rectal surgery 2009;22:156-60.

6. Friend K, Mills AM. Is outpatient oral antibiotic therapy safe and effective for the treatment of acute uncomplicated diverticulitis? Annals of Emergency Medicine 2011;57:600-2.

7. Gargallo Puyuelo CJ, Sopena F, Lanas Arbeloa A. Colonic diverticular disease. Treatment and prevention. Gastroenterologia y Hepatologia 2015;38:590-9.

8. Genser L, Karoui M, Vaillant JC, Hannoun L. [Medical treatment of sigmoid diverticulitis]. La Revue du praticien 2013;63:821, 5-6.

9. Isacson D, Andreasson K, Nikberg M, Smedh K, Chabok A. Changed treatment strategy in acute uncomplicated diverticulitis resulted in hospital bed and health care cost savings: A population based study. Colorectal Disease 2016;18:7.

10. Kohn MA. Outpatient versus hospitalization management for uncomplicated diverticulitis. Annals of Surgery 2015;262:e86.

11. Lombardo L, Lapertosa G. [The ambulatory medical treatment of colonic diverticulitis. An open clinico-endoscopic-histological study with rifaximin, a nonaminoglycoside enteric antibiotic]. Recenti progressi in medicina 1991;82:300-4.

12. Mizuki A, Nagata H, Tatemichi M, et al. The out-patient management of patients with acute mild-to-moderate colonic diverticulitis. Alimentary Pharmacology and Therapeutics 2005;21:889-97.

13. Mora Lopez L, Serra Pla S, Serra-Aracil X, Ballesteros E, Navarro S. Application of a modified Neff classification to patients with uncomplicated diverticulitis. Colorectal Disease 2013;15:1442-7.

14. Paolillo C, Spallino I. Is it safe to send home an uncomplicated diverticulitis? The DIVER trial. Internal and emergency medicine 2015;10:193-4.

15 Papagrigoriadis S, Debrah S, Koreli A, Husain A. Impact of diverticular disease on hospital costs and activity. Colorectal Disease 2004;6:81-4.

16. Ribas Y, Aguilar F, Bargallo J, Lamas S, Campillo F. Medical treatment for uncomplicated acute diverticulitis: are we being efficient and effective enough? Int J Colorectal Dis 2013;28:729-30.

17. Ridgway PF, Latif A, Shabbir J, et al. Randomized controlled trial of oral vs intravenous therapy for the clinically diagnosed acute uncomplicated diverticulitis. Colorectal Disease 2009;11:941-6.

18. Salem TA. Course is benign for uncomplicated diverticular disease. Journal of Family Practice 2007;56:992.

19. Schmidt-Lauber M. Outpatient treatment for uncomplicated diverticulitis. [German]. Coloproctology 2015;37:285-6.

20. Steurer J. [Outpatient treatment of patients with acute uncomplicated diverticulitis]. Praxis 2013;102:1388-9.

21. Tursi A. Efficacy, safety, and applicability of outpatient treatment for diverticulitis. E-mail: angela@dovepress.com: Dove Medical Press; 2014:29-36.
