# Supplementary material for: A systematic review and meta-analysis of outpatient treatment for acute diverticulitis
Source: Int J Colorectal Dis. 2018 Mar 12;33(5):505–12. doi: 10.1007/s00384-018-3015-9 (PMC5899114; doi:10.1007/s00384-018-3015-9)
Supplement: Supplementary file 4 — Evidence table. (DOCX 134 kb) [file 384_2018_3015_MOESM4_ESM.docx]

**Online Resource 4. Evidence table.**

| **Study** | | **Selection and definition** | | | | | |  | |
| --- | --- | --- | --- | --- | --- | --- | --- | --- | --- |
| **Study** | **Design** | **Diag-nosis** | **Left-sided diverticulitis** | **Inclusion criteria** | **Exclusion criteria** | **Outpatient treatment** | **Criteria for assignment to group** | **Outcome** | **Results** |
| **Alonso** 2010 | Pro-spective cohort | CT | 100% | No abscess, free air or generalized ascites on CT | Inability to tolerate oral intake, comorbidity (diabetes mellitus, heart failure, renal insufficiency, chronic obstructive pulmonary disease), lack of adequate family or social support. | Oral antibiotics, follow-up outpatient clinic after 4-7 days | Only outpatient group | Readmission | 3% (2/70) |
|  |  |  |  |  |  |  |  | Need for emergency surgery | 0% (0/70) |
|  |  |  |  |  |  |  |  | Need for percutaneous abscess drainage | 0% (0/70) |
| **Biondo** 2014 | RCT | CT | 100% | Modified Hinchey stage 1a on CT, able to tolerate oral intake, good response to first treatment measures in emergency department (improvement of pain, fever and/or tenderness), willing to continue treatment at home under supervision. | Pregnancy or breastfeeding, intake of antibiotic for colonic diverticulitis in the month previous to actual diagnosis, colorectal cancer suspicion at CT, concomitant unstable comorbid conditions, immunosuppression, cognitive social or psychiatric impairment, intolerance to oral intake and persisting vomiting, patients' rejection of written consent. | Oral antibiotics, daily contact by telephone for 5 consecutive days by the study investigator, appointment with an investigator physician close to day 14 after discharge in the outpatient clinic. | Assignment based on randomization | Readmission | Out: 4.5% (3/66)  In: 6.1% (4/66) |
|  |  |  |  |  |  |  |  | Need for emergency surgery | Out: 0.0% (0/66)  In: 0.0% (0/66) |
|  |  |  |  |  |  |  |  | Need for percutaneous abscess drainage | Out: 0.0% (0/66)  In: 0.0% (0/66) |
|  |  |  |  |  |  |  |  | Costs per patient | Out: €547.05  In: €1671.75 |
| **Estrada** 2016 | Pro-spective cohort | CT | 100% | Modified Neff classification 0 on CT, no acute diverticulitis episode in the last 3 months, immunocompetent, no significant comorbidities (diabetes mellitus, renal insufficiency, morbid obesity), age <80 years, good oral tolerance, good symptom control by oral medication, adequate family support, patients' written informed consent, maximum one of SIRS criteria or CRP >15. | Mild diverticulitis patients that received at least one dose of antibiotic in the emergency department. | No antibiotics, review in the surgical outpatients clinic after 48h and 7 days. | All outpatients met in- and exclusion criteria, all inpatients did not | Readmission | Out: 11.1% (4/36)  In: 33.3% (3/9) |
|  |  |  |  |  |  |  |  | Need for emergency surgery | Out: 0.0% (0/36)  In: 0.0% (0/9) |
|  |  |  |  |  |  |  |  | Need for percutaneous abscess drainage | Out: 0.0% (0/36)  In: 0.0% (0/9) |
| **Etzioni** 2010 | Retro-spective cohort | CT | Not reported | Patients with a primary episode of acute diverticulitis that received outpatient treatment. Criteria for outpatient treatment were not mentioned. | Patients that were admitted for inpatient treatment on the day of ED evaluation or the next calendar day, prior diagnosis of colorectal cancer or inflammatory bowel disease, treatment of diverticulitis episode without antibiotics. | Outpatient treatment not specified. | Only outpatient group | Non-elective readmission or emergency department evaluation within 60 days after the date of the index ED evaluation | 5.6% (39/693) |
| **Isacson** 2015 | Pro-spective cohort | CT | 100% | Modified Hinchey 1a on CT, written informed consent. | High fever, affected general condition, peritonitis, septicaemia, ongoing antibiotic therapy, dehydrated patient or persistent vomiting in need of intravenous fluid administration, pain requiring intravenous or subcutaneous morphine, immunologically comprised patients, pregnancy, dementia, patients with language barriers, patients who cannot take care of themselves at home or are unable to follow instructions. | No antibiotics, daily contact via telephone by a nurse, surgical clinic visit after 1 week and 3 months. | Only outpatient group | Readmission | 2.6% (4/155) |
|  |  |  |  |  |  |  |  | Need for emergency surgery | 0.0% (0/155) |
|  |  |  |  |  |  |  |  | Need for percutaneous abscess drainage | 0.6% (1/155) |
| **Joliat**  2017 | Retro-spective cohort | CT | 96% | Modified Hinchey 1a, pneumoperi-toneum <2cm or abscess <4cm. Responded to questionnaire. | Patients requiring an immediate (at admission) percutaneous drainage or surgery. | Oral antibiotics. | Assignment based on decision of attending physician. | Readmission | Out: 10.2% (10/98)  In: 32.0% (54/169) |
| **Lorente** 2013 | Retro-spective cohort | CT | Not reported | Modified Hinchey 1a on CT, tolerance to oral intake prior to discharge from the ED, absence of comorbidities and adequate family of social support. | None | Oral antibiotics, outpatient clinic visit 4-7 days after diagnosis | Assignment based on decision of attending physician. | Readmission | Out: 5.6% (5/90)  In: 4.3% (2/46) |
|  |  |  |  |  |  |  |  | Need for emergency surgery | Out: 0.0% (0/90)  In: 0.0 (0/46) |
|  |  |  |  |  |  |  |  | Need for percutaneous abscess drainage | Out: 0.0% (0/90)  In: 0.0 (0/46) |
|  |  |  |  |  |  |  |  | Costs per patient | Out: €882.00  In: €2376.00 |
| **Lutwak** 2012 | Retro-spective cohort | CT | Not reported | Uncomplicated diverticulitis without abscess on CT, reliable, able to tolerate the oral medication, lacked significant co-morbidities, adequate social support. | Patients in septic shock, hemodynamically comprised, generalized peritonitis, requiring aggressive surgical treatment. | Oral antibiotics. | Assignment based on decision of attending physician. | Readmission | Out: 14.3% (3/21)  In: 0.0% (0/21) |
|  |  |  |  |  |  |  |  | Need for emergency surgery | Out: 0.0% (0/21)  In: 0.0% (0/21) |
|  |  |  |  |  |  |  |  | Need for percutaneous abscess drainage | Out: 0.0% (0/21)  In: 0.0% (0/21) |
| **Mali** 2016 | Pro-spective cohort | CT | 94% | No abscess, fistula or obstruction, free air in the abdominal cavity or retroperitoneal air on CT, pericolic air was allowed. | Ongoing treatment with antibiotics, immunosuppressive state (including diabetes mellitus, chemotherapy or chronic liver disease), suspicion of peritonitis, organ failure, another infection requiring treatment with antibiotics, pregnancy, >90 years old, missing signed informed consent. | No antibiotics, clinical control after 24 to 48h. | Assignment based on decision of attending physician. | Readmission | Out: 2.9% (4/140)  In: not reported |
|  |  |  |  |  |  |  |  | Need for emergency surgery | Out: 0.0% (0/140)  In: 0.0% (0/21) |
| **Martin Gil** 2009 | Pro-spective cohort | CT | Not reported | Hinchey 1 diverticulitis and a selected group of Hinchey 2 patients (abscess size <3cm, clinically and biochemically stable). | Immunosuppressed patients | Observation for 24 hours in the emergency room with nil-per-os diet and intravenous antibiotics, discharge from the ED with oral antibiotics, outpatient clinic visit 10 days after diagnosis. | Only outpatient group | Readmission | 5.4% (4/74) |
|  |  |  |  |  |  |  |  | Need for emergency surgery | 0.0% (0/74) |
|  |  |  |  |  |  |  |  | Need for percutaneous abscess drainage | 0.0% (0/74) |
|  |  |  |  |  |  |  |  | Costs per patient | Out: €1280.00  In: €2192.00 |
| **Mora** 2017 | Pro-spective cohort | CT | Not reported | Modified Neff classification 0 = diverticulitis without abscess or distant free air, comfortable enough and able to tolerate liquids. | Diabetes mellitus, alcoholism, liver disease, heart disease, neoplastic disease, inflammatory intestinal disease, previous hospitalization in the last 30 days, age >80 years, lack of family support, one of the SIRS criteria | Oral antibiotics, surgical outpatient clinic visit after 2 weeks | All outpatients met in- and exclusion criteria, all inpatients did not | Readmission | 8.7% (22/254) |
|  |  |  |  |  |  |  |  | Need for emergency surgery | Out: 0.0% (0/254)  In: 2.8%  (5/176) |
|  |  |  |  |  |  |  |  | Need for percutaneous abscess drainage | 0.0% (0/254) |
| **Moya** 2012 | Pro-spective cohort | CT | Out: 84%  In: 91% | Diverticulitis without abundant free fluid, intra-abdominal abscess or pneumoperitoneum on CT, age <90 years, immunocompetent patient, tolerating oral intake, no signs of severe sepsis, adequate family or social support network. | None | Oral antibiotics, clinical checks and tests at 4 days and 7 days. | Assignment to outpatient or inpatient group based on time period, between January 2007 and May 2008 as inpatients and between May 2008 and December 2009 as outpatients. | Readmission | Out: 6.3% (2/32)  In: 0.0% (0/44) |
|  |  |  |  |  |  |  |  | Need for emergency surgery | Out: 0.0% (0/32)  In: 0.0% (0/44) |
|  |  |  |  |  |  |  |  | Need for percutaneous abscess drainage | Out: 0.0% (0/32)  In: 0.0% (0/44) |
|  |  |  |  |  |  |  |  | Costs per patient | Out: €347.31  In: €1945.26 |
| **Moya** 2016 | Retro-spective cohort | CT | 95% | Peridiverticulitis or abscess <3cm on CT, age <90 years, immunocompetent, tolerated oral intake, no signs of severe sepsis, adequate family and social support network. | None | Oral antibiotics, clinical checks at 4 days and 7 days. | Only outpatient group | Readmission | 8.0% (18/224) |
|  |  |  |  |  |  |  |  | Need for emergency surgery | 0.0% (0/224) |
|  |  |  |  |  |  |  |  | Need for percutaneous abscess drainage | 0.0% (0/224) |
| **Pelaez** 2006 | Pro-spective cohort | CT | 100% | Uncomplicated diverticulitis without abscess on CT. | Vomiting, major comorbidities (diabetes mellitus, heart failure, renal failure), no family or social support. | Oral antibiotics, outpatients clinic visit at 4 and 7 days after diagnosis | Only outpatient group | Readmission | 5.0% (2/40) |
|  |  |  |  |  |  |  |  | Need for emergency surgery | 0.0% (0/40) |
|  |  |  |  |  |  |  |  | Need for percutaneous abscess drainage | 0.0% (0/40) |
| **Rodriguez** 2010 | Pro-spective cohort | CT | Not reported | No abscess or perforation, phlegmon, fistula or obstruction on CT, care giver 24h a day, absence of immunosuppressive therapy, acceptance of home treatment, improvement clinical condition in observation ward, present comorbidity (cardiopathy, diabetes mellitus, chronic renal failure). | None | Treatment in Hospital at Home Unit after a 12-18h observation at the Emergency Department Observation Ward, treatment at patient's home with intravenous antibiotics until their clinical condition improved after which the antibiotics were administered orally, physician visits 2-3 per week, nurse daily visits | Only outpatient group | Readmission | 0.0% (0/24) |
|  |  |  |  |  |  |  |  | Need for emergency surgery | 0.0% (0/24) |
|  |  |  |  |  |  |  |  | Need for percutaneous abscess drainage | 0.0% (0/24) |
| **Rodriguez** 2013 | Pro-spective cohort | CT | Not reported | No peritonitis or abscess, fistula or obstruction on CT, age > 70 years, care giver 24h a day, willing to be treated at home. | Clinical worsening in the observation ward of the emergency department,  β-lactam allergy | Treatment in Hospital at Home Unit after a 24h observation at the Emergency Department Observation Ward, treatment at patient's home with intravenous antibiotics until 4-6 days after which the antibiotics were administered orally, physician visits 2-3 per week, nurse daily visits. | All outpatients met in- and exclusion criteria, all inpatients did not | Readmission | 0.0% (0/34) |
|  |  |  |  |  |  |  |  | Need for emergency surgery | 0.0% (0/34) |
|  |  |  |  |  |  |  |  | Need for percutaneous abscess drainage | 0.0% (0/34) |
|  |  |  |  |  |  |  |  | Costs per patient | Outpatient €1368.00 cheaper than inpatient, absolute costs not reported |
| **Rueda** 2012 | Retro-spective cohort | CT | Not reported | Hinchey 1 or 2 on CT, patients younger than 80 years of age who presented in good general health, ASA 1 or 2, absence of clinical signs of complications such as peritonitis, vomiting or severe abdominal distension, family could provide proper environment, the patient agreed to receive home care. | None | Treatment in the Home Care Unit, home treatment but not specified, treatment with intravenous antibiotics | All outpatients met inclusion criteria, all inpatients did not | Readmission | Out: 21.1% (8/38)  In: 27.8% (5/18) |
| **Sirany**  2017 | Retro-spective cohort | CT | 96% | Modified Hinchey 1a, pericolic extraluminal air, diverticular abscess | Patients without follow-up after the index encounter, previous colonic resection, history of inflammatory bowel disease. | Oral antibiotics. | Assignment based on decision of attending physician. | Readmission | Out: 12.5% (12/96)  In: 15.3% (22/144) |
|  |  |  |  |  |  |  |  | Need for emergency surgery | Out: 1.0% (1/96)  In: 6.9% (10/144) |
|  |  |  |  |  |  |  |  | Need for percutaneous abscess drainage | Out: 0.0% (0/96)  In: 4.2% (6/144) |
| **Ünlü** 2013 | Retro-spective cohort | CT or sono-grap-hy | 100% | Hinchey 1 on ultrasound or CT | Recurrent diverticulitis, right sided diverticulitis, incomplete follow-up. | Hospital admission less than 24h, all managed in the emergency department, outpatient clinic visit after 1 week. | Assignment based on decision of attending physician. | Readmission | Out: 8.5% (10/118)  In: not reported |
|  |  |  |  |  |  |  |  | Need for emergency surgery | Out: 0.8% (1/118)  In: 1.5% (3/194) |
|  |  |  |  |  |  |  |  | Need for percutaneous abscess drainage | Out: 0.0% (0/118)  In: 0.5% (1/194) |
