# Supplementary material for: A systematic review and meta-analysis of outpatient treatment for acute diverticulitis
Source: Int J Colorectal Dis. 2018 Mar 12;33(5):505–12. doi: 10.1007/s00384-018-3015-9 (PMC5899114; doi:10.1007/s00384-018-3015-9)
Supplement: Supplementary file 5 — Risk of bias table of randomized clinical trial Biondo et al. [17] (DOCX 31 kb) [file 384_2018_3015_MOESM5_ESM.docx]

**Online Resource 5. Risk of bias table of randomized clinical trial Biondo et al.^17^**

| **Entry** | **Judgement** | **Support for judgement** |
| --- | --- | --- |
| Random sequence generation (selection bias) | Low risk | “Randomization was performed by using a computer-generated random code and stratified by centre. The random code was held centrally in a sealed envelope and distributed to each centre by the monitor of the study.” |
| Allocation concealment (selection bias) | Unclear risk | No information whether envelopes were sequentially numbered or opaque. |
| Blinding of participants and personnel (performance bias) | High risk | No blinding of participants and personnel. |
| Blinding of outcome assessment (detection bias) | Low risk | No blinding but outcome not likely to be influenced. |
| Incomplete outcome data addressed (detection bias) | Low risk | In both group one patient lost to follow-up, in the control group 1 protocol violation and in the intervention group 2 adverse events. |
| Selective reporting (reporting bias) | Low risk | No protocol available but all relevant outcomes reported. |
