# Supplementary material for: A systematic review and meta-analysis of outpatient treatment for acute diverticulitis
Source: Int J Colorectal Dis. 2018 Mar 12;33(5):505–12. doi: 10.1007/s00384-018-3015-9 (PMC5899114; doi:10.1007/s00384-018-3015-9)
Supplement: Supplementary file 6 — Newcastle Ottawa risk of bias table of observational cohort studies. (DOCX 54 kb) [file 384_2018_3015_MOESM6_ESM.docx]

**Online Resource 6. Newcastle Ottawa risk of bias table of observational cohort studies.**

| ***Observational cohort studies***  **Author + year** | **Representative-ness of cohort** | **Selection of non-exposed cohort** | **Ascertainment of exposure** | **Demonstration that outcome of interest was not present at start** | **Comparability of cohorts** | **Assessment of outcome** | **Follow up long enough** | **Adequacy of follow up of cohorts** | **Total** |
| --- | --- | --- | --- | --- | --- | --- | --- | --- | --- |
| **Alonso** 2010 | A* | C | A* | A* | - | B* | A* | B* | 6 |
| **Estrada** 2016 | A* | B | A* | A* | - | B* | A* | A* | 6 |
| **Etzioni** 2010 | A* | C | A* | A* | - | B* | A* | D | 5 |
| **Isacson** 2015 | A* | C | A* | A* | - | B* | A* | B* | 6 |
| **Joliat** 2017 | B* | B | A* | A* | - | C | A* | C | 4 |
| **Lorente** 2013 | A* | B | A* | A* | - | B* | A* | D | 5 |
| **Lutwak** 2012 | A* | B | A* | A* | - | B* | A* | A* | 6 |
| **Mali** 2016 | A* | C | A* | A* | - | B* | A* | B* | 6 |
| **Martin Gil** 2009 | A* | C | A* | A* | - | B* | A* | C | 5 |
| **Mora** 2017 | A* | C | A* | A* | - | B* | A* | D | 5 |
| **Moya** 2012 | A* | A* | A* | A* | - | B* | A* | A* | 7 |
| **Moya** 2016 | A* | C | A* | A* | - | B* | A* | A* | 6 |
| **Pelaez** 2006 | A* | C | A* | A* | - | B* | A* | D | 5 |
| **Rodriguez** 2010 | C | C | A* | A* | - | B* | A* | D | 4 |
| **Rodriguez** 2013 | C | C | A* | A* | - | B* | A* | D | 4 |
| **Rueda** 2012 | B* | B | A* | A* | - | B* | A* | D | 5 |
| **Sirany** 2017 | A* | B | A* | A* | - | B* | A* | A* | 6 |
| **Ünlü** 2013 | A* | C | A* | A* | - | B* | A* | A* | 6 |
