# Supplementary figures and images for: A systematic review and meta-analysis of outpatient treatment for acute diverticulitis
Source: Int J Colorectal Dis. 2018 Mar 12;33(5):505–12. doi: 10.1007/s00384-018-3015-9 (PMC5899114; doi:10.1007/s00384-018-3015-9)

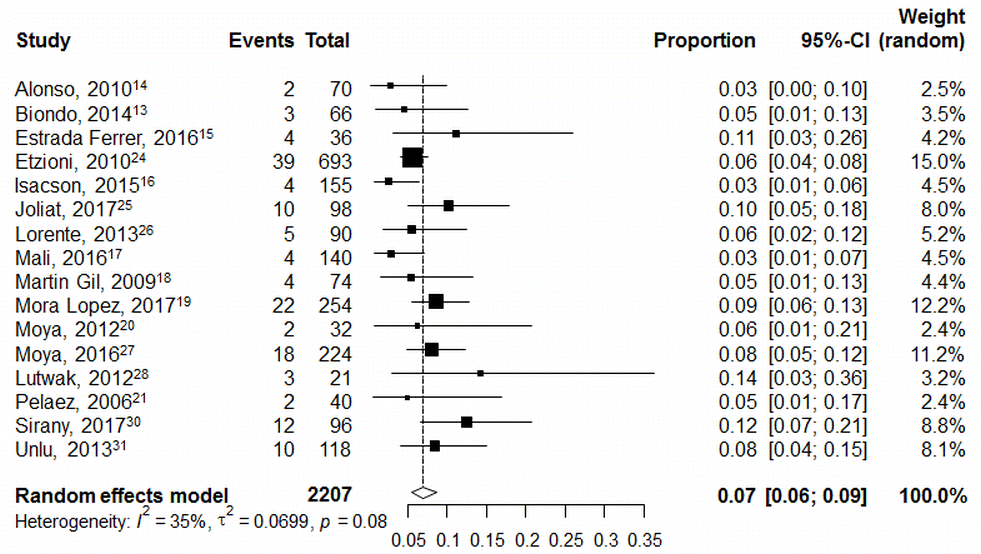

Supplement: Supplementary file 7 — Forest plot of pooled incidence rate of readmission in patients that received outpatient treatment excluding 3 studies that employed a deviated protocol [26, 27, 33] .(GIF 165 kb) [file 384_2018_3015_Fig3_ESM.gif]

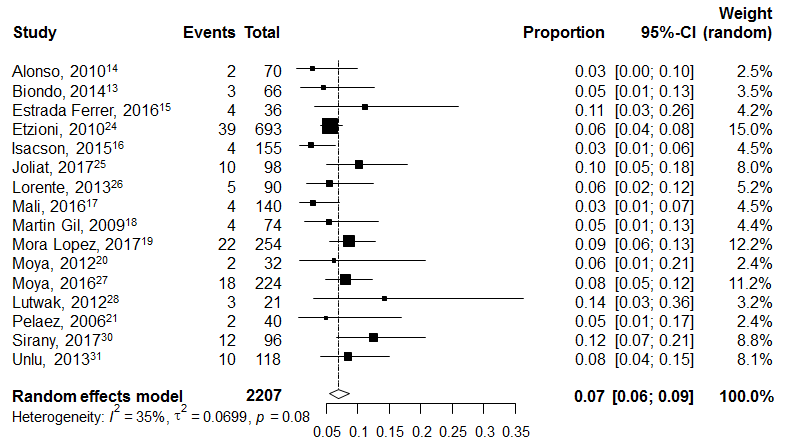

Supplement: Supplementary file 8 — High resolution image (TIFF 53 kb) [file 384_2018_3015_MOESM7_ESM.tif]

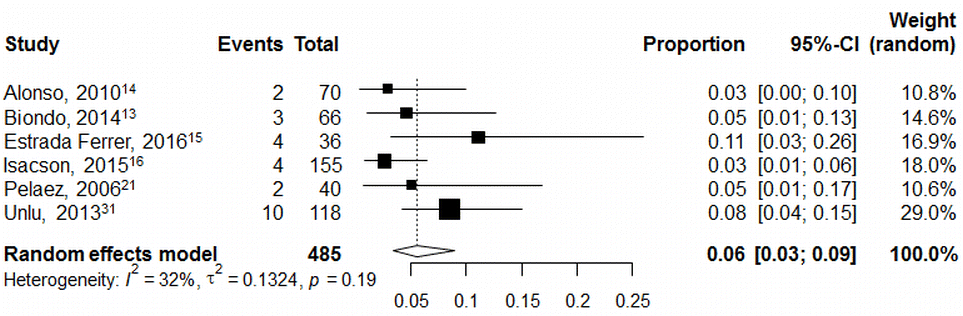

Supplement: Supplementary file 9 — Forest plot of pooled incidence rate of readmission in patients that received outpatient treatment only from studies with confirmed 100% left-sided diverticulitis. (GIF 88 kb) [file 384_2018_3015_Fig4_ESM.gif]

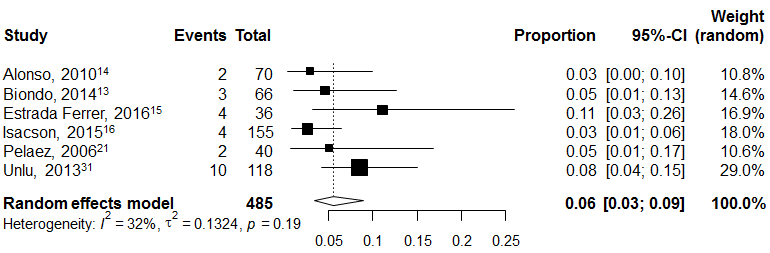

Supplement: Supplementary file 10 — High resolution image (TIFF 28 kb) [file 384_2018_3015_MOESM8_ESM.tif]
